# Early Embryonic Gene Expression Profiling of Zebrafish Prion Protein (Prp2) Morphants

**DOI:** 10.1371/journal.pone.0013573

**Published:** 2010-10-22

**Authors:** Rasoul Nourizadeh-Lillabadi, Jacob Seilø Torgersen, Olav Vestrheim, Melanie König, Peter Aleström, Mohasina Syed

**Affiliations:** Department of Basic Sciences and Aquatic Medicine, Norwegian School of Veterinary Science, Oslo, Norway; University of Canterbury, New Zealand

## Abstract

**Background:**

The Prion protein (PRNP/Prp) plays a crucial role in transmissible spongiform encephalopathies (TSEs) like Creutzfeldt-Jakob disease (CJD), scrapie and mad cow disease. Notwithstanding the importance in human and animal disease, fundamental aspects of PRNP/Prp function and transmission remains unaccounted for.

**Methodology/Principal Findings:**

The zebrafish (*Danio rerio*) genome contains three Prp encoding genes assigned *prp1*, *prp2 and prp3*. Currently, the second paralogue is believed to be the most similar to the mammalian *PRNP* gene in structure and function. Functional studies of the *PRNP* gene ortholog was addressed by *prp2* morpholino (MO) knockdown experiments. Investigation of Prp2 depleted embryos revealed high mortality and apoptosis at 24 hours post fertilization (hpf) as well as impaired brain and neuronal development. In order to elucidate the underlying mechanisms, a genome-wide transcriptome analysis was carried out in viable 24 hpf morphants. The resulting changes in gene expression profiles revealed 249 differently expressed genes linked to biological processes like cell death, neurogenesis and embryonic development.

**Conclusions/Significance:**

The current study contributes to the understanding of basic Prp functions and demonstrates that the zebrafish is an excellent model to address the role of Prp in vertebrates. The gene knockdown of *prp2* indicates an essential biological function for the zebrafish ortholog with a morphant phenotype that suggests a neurodegenerative action and gene expression effects which are apoptosis related and effects gene networks controlling neurogenesis and embryo development.

## Introduction

The molecular mechanisms responsible for prion pathogenesis share similarities with a wider group of neurodegenerative disorders. The genetic tractability of zebrafish (*Danio rerio*) makes it a suitable model for addressing the fundamental molecular mechanisms underlying neurodegenerative disorders such as prion disease [1], Alzheimer disease [2] and Parkinson's disease [3].

In the prion theory of Prusiner [1] transmissible spongiform encephalopathies are proposed to be caused by a proteinaceous infectious agent, a pathological variant of the normal cellular prion protein, PRNP scrapie (PRNP^Sc^; also named PrP^Sc^), which acts as a template for the conversion of normal cellular prion protein PRNP^C^ (also named PrP^C^) to PRNP^Sc^. There are however, major unresolved issues in prion research relating to the more fundamental biological questions of the prion gene function and mechanisms for transmission of the disease. PRNP is a glycosylated glycosylphosphatidylinositol (GPI) anchored membrane protein, but its function, cellular distribution and polarized sorting in the cell remain to be definitively established. Also the functional relationship with its binding partner(s) remains unaccounted for. A list of PRNP^C^ candidate binding partner proteins together with the major proposed functions for PRNP^C^ itself has been summarized in a review by Westergard *et al.* [4], including protection against apoptosis and oxidative stress, copper ions uptake and binding, synaptic signal transduction and as linker to the extracellular matrix.

The PRNP gene is phylogenetically conserved, though teleost genomes harbor multiple *prp* paralogues due to extra genome duplication. Of the three zebrafish *PRNP* genes, *prp3* is the more divergent variant [5], whereas *prp1* and *prp2* more closely resemble the mammalian variant with *prp2* most likely being the closest ortholog [6]. Miesbauer *et al.* [7] demonstrated that PrP-related proteins from zebrafish are complexly glycosylated and contain a glycosylphosphatidylinositol anchor. It appears that the nomenclature for prion protein variants and the corresponding genes can be confusing (Table S1). In this paper we have chosen to use *prp1*, *prp2*, *prp3* and Prp1, Prp2, Prp3 for the zebrafish genes and proteins respectively. For the mammalian homolog we denote the normal variant gene and protein *PRNP* and PRNP (PrP^C^) respectively. The misfolded pathological PRNP scrapie variant is termed PRNP^Sc^ (PrP^Sc^).

Knockout mice lacking the neuronal cell surface PRNP protein show normal development and behavior [8]. On the contrary, *prp1* mRNA knockdown in zebrafish results in a strong embryonal phenotype, characterized by absence of embryonic cell adhesion and arrested gastrulation [9]. Similarly, *prp2* morphant zebrafish also developed a lethal phenotype but at a later larval stage at 7 days post-fertilization (dpf) [9]. In the same study it was also demonstrated that both zebrafish Prp and mouse PRNP mRNAs could rescue the knockdown phenotype, indicating an evolutionary conserved Prion protein function.

The apparent difference between the mouse gene knockout and zebrafish models suggests that important aspects of the Prion protein biology may be revealed in the zebrafish model, using morpholino (MO) mediated translation blocking [10]. Aiming at uncovering the function of zebrafish *prp2* gene, two MOs (prp2-MO1 and prp2-MO2) for targeting the *prp2* mRNA were designed and injected into one cell stage zebrafish embryos. The resulting phenotypes were characterized and microarray studies were conducted on 24 hpf zebrafish embryos microinjected with the most potent of the two MOs, prp2-MO2. In order to confirm the microarray data and demonstrate that both MOs showed the same specificity, quantitative real-time PCR (qRT-PCR) was performed on mRNA of selected genes from both prp2-MO1 and prp2-MO2 injected embryos.

## Results

**Figure 1:**
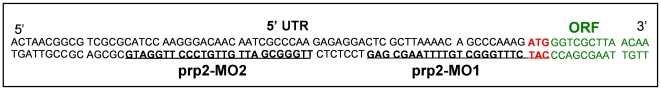
Morpholino sequences. Morpholino sequences (prp2-MO1and prp2-MO2 sequences underlined) used for knockdown of the zebrafish PrP2 gene function.

**Figure 2:**
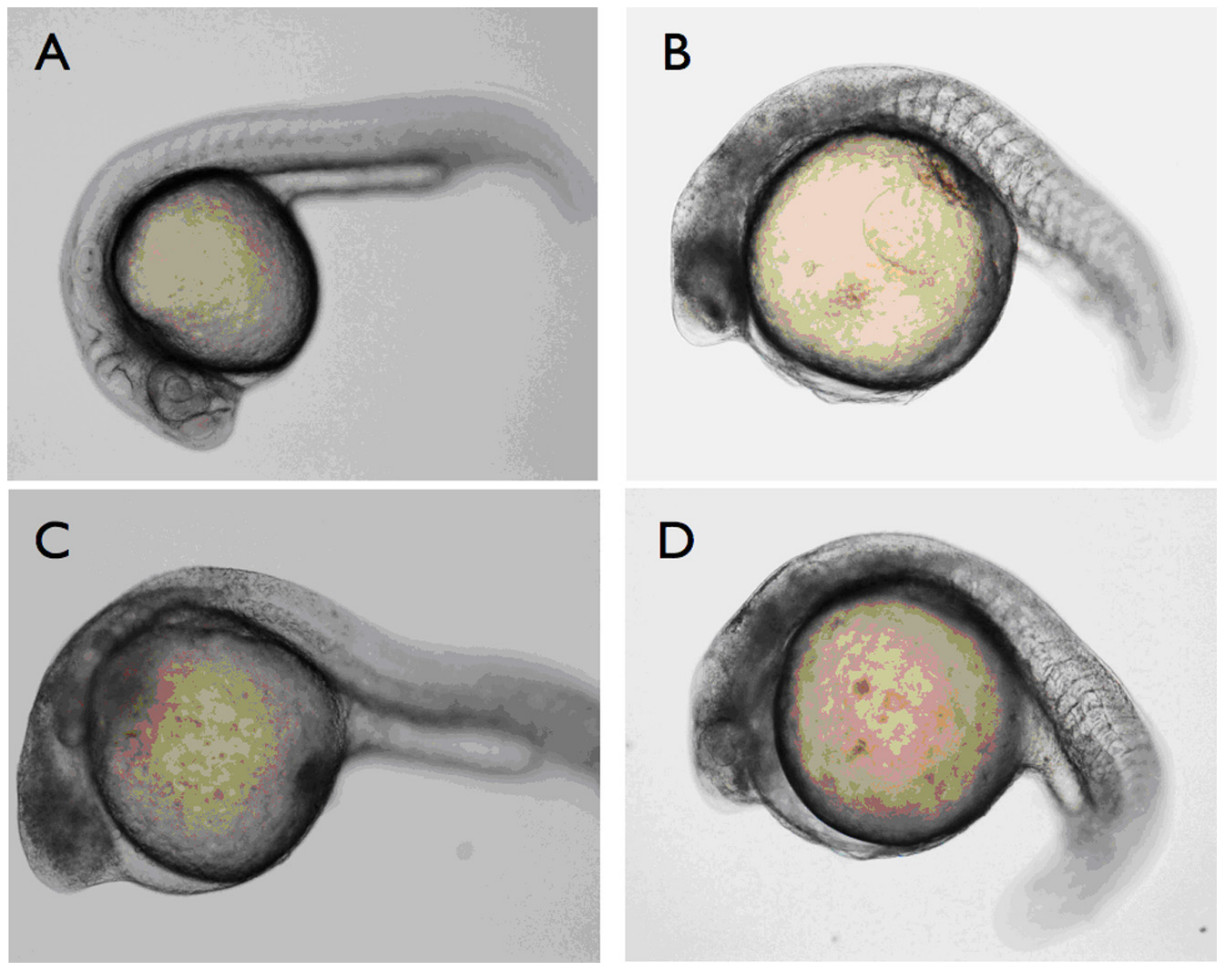
prp2-MO2 morphant 24 hpf larvae phenotypes. Larvae were immobilized in CyGEL prior to microscopy. **A**) Non-injected wt control. **B–D**) three individual morphants displaying defective midbrain and hindbrain development.

**Figure 3:**
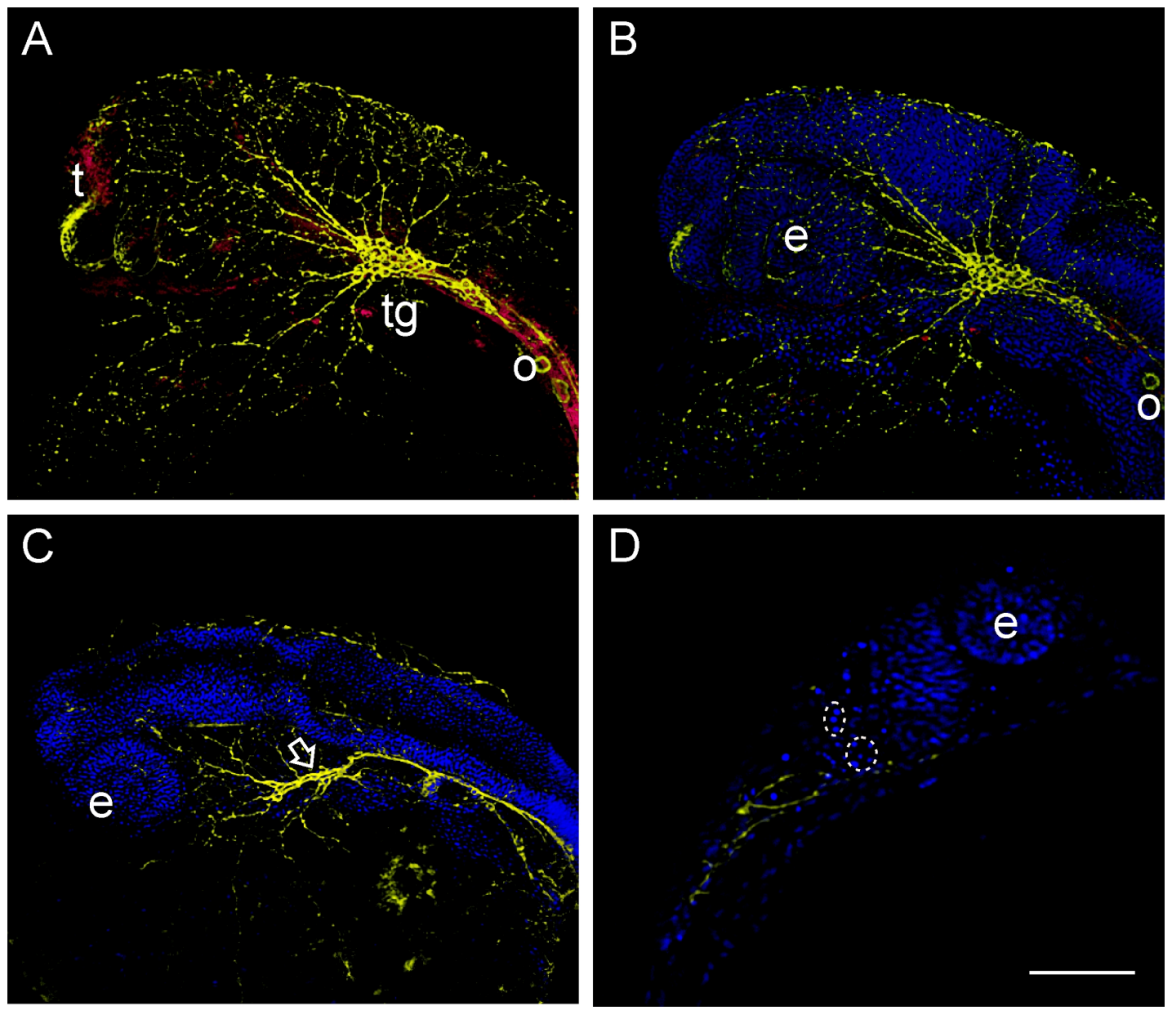
Whole mount immunofluorescence analysis of 24 hpf wild type (A and B) and Prp2 morphants (C and D). Neuronal structures were visualized by the Zn12 antibody (Yellow), whereas Prp2 was detected with a rabbit anti serum (Red). **A**) In the wild type 24 hpf Prp2 is observed in the trigeminal ganglion and telencephalon, but also present in other neuronal tissue. **B**) Neuronal structures visualized by HNK-1 staining (Zn-12 antibody) in the same specimen as in A. **C**) Aberrant morphology of the trigeminal ganglion in Prp2 morphants (arrow) as well as reduced number of peripheral neurons visualized by HNK-1 staining. **D**) Non deconvolved single plane image of a morphant with condensed nuclei (inside stippled ring). Abbreviations: eye (e), telencephalon (t) and trigeminal ganglion (tg).

Optimization of the two MOs specific for the 5′UTR of GenBank sequence AJ620614.1 (Fig. 1) injections revealed that 0.2 mM prp2-MO2 resulted in 71% mortality at 24 hpf and 74% at 48 hpf. In order to reduce the mortality, 0.15 mM prp2-MO2 was chosen because it led to a mortality rate below 50%. Phase contrast microscopy of Prp2 morphants revealed a clear phenotype with midbrain and hindbrain developmental defects as compared to the control group (Fig. 2 B–D). In order to further characterize the phenotypic effects, wholemount immunofluorescence was carried out using antibodies against Prp2 and HNK-1. Fluorescence microscopy analysis of the wild type embryos at 24 hpf unveiled low but significant levels of Prp2 proteins in the telencephalon, eye and trigeminal ganglion (Fig. 3A). Investigation of HNK-1 showed strong staining to the trigeminal ganglion and neuronal extensions (Fig. 3B). In the morphants no Prp2 activity could be detected and the Zn12 antibody visualized altered trigeminal ganglion morphology and reduced number of peripheral neurons in the head (Fig. 3C). Also, nuclear staining with DAP1 revealed apoptotic cells with hyper-condensed nuclei in the trigeminal ganglion, eye, olfactory placode, and in the midbrain-hindbrain area (Fig. 3D).

**Figure 4:**
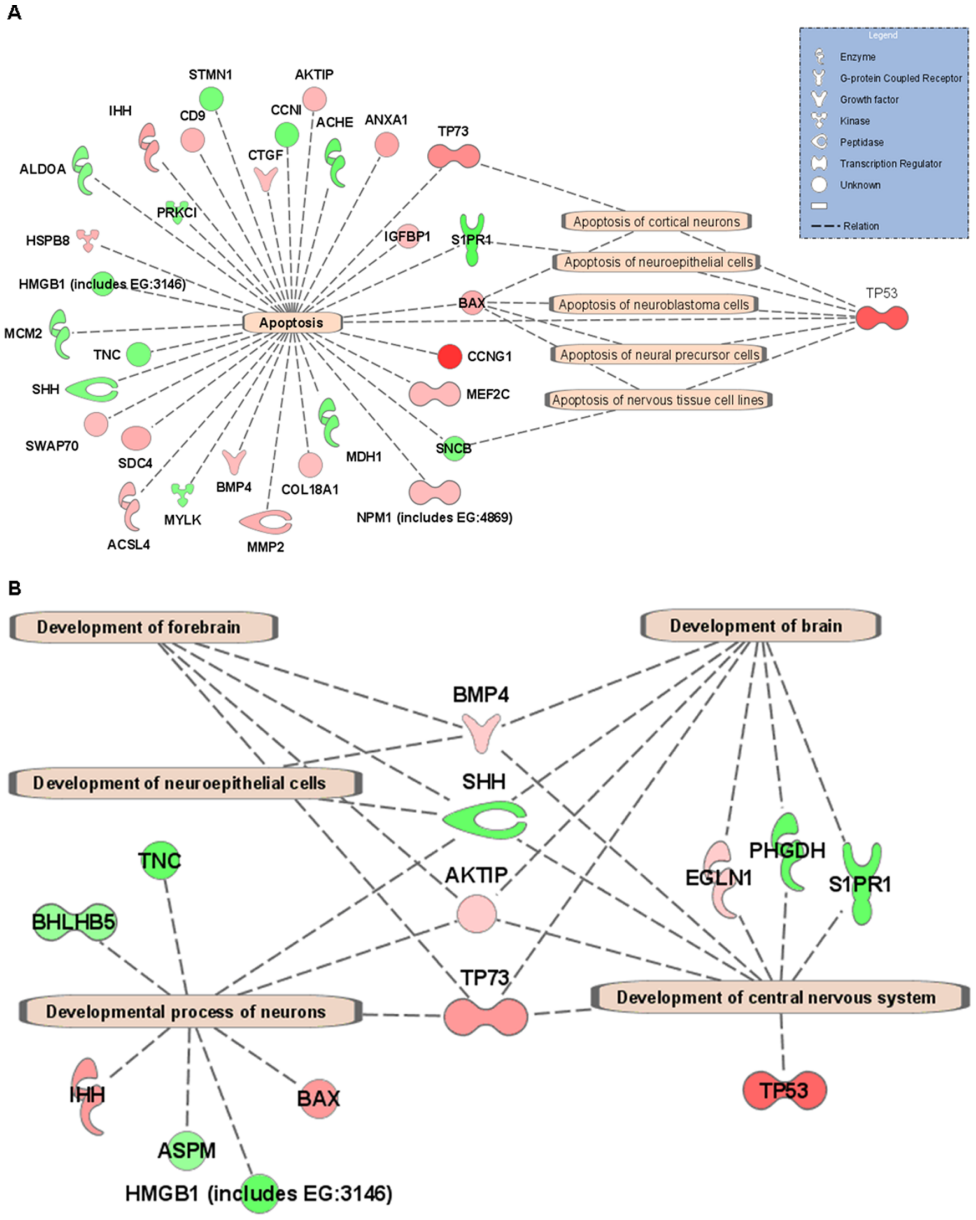
IPA cluster analysis of significantly differentially expressed genes. IPA cluster analysis of significantly differentially expressed genes for 24 hpf zebrafish prp2-MO2 morphant embryos. The clusters reveal genes involved in (**A**) cell death and apoptosis with functions focused on apoptosis in neural cells and (**B**) genes involved in nervous system development. Red color indicates up- and green downregulation.

Microarray analysis using a 16k oligonucleotide library revealed that 249 genes were significantly differentially expressed in the prp2-MO2 samples (Table 1). IPA analysis identified and mapped 120 of the 249 genes as being genes with mammalian ortholog identifiers (Table S2). The remaining 129 genes were either zebrafish specific or yet non-annotated genes with hypothetical or unknown function (Table S3). The most upregulated gene was cyclin G1 (*CCNG1*) with a 4.2 fold change. Leucine rich repeat neuronal 1 gene (*LPRN1*) was the most downregulated gene with a minus 2.46 fold change. The majority of the differentially expressed genes were encoding enzymes or transcription regulators with their location in the cytoplasm or nucleus, respectively. The IPA organization of differentially expressed genes into biological function and disease clusters included “cell death”, “embryonic development” and “nervous system development and function” (Fig. S1, Table 2). The “cell death/apoptosis” related gene cluster was the largest sub-cluster with a total of 32 genes, of which 5 were related to apoptosis of neural cells: *TP53*, *TP73*, and *BAX* (upregulated) and *SNPR1* and *SNCB* (downregulated, Fig. 4A, Table S2). The cluster of “embryonic development” (Fig. S1, Table 2) contained 11 genes, of which IPA could join 9 to each other in a network of sub-clusters in which *SHH* was the only downregulated gene and BMP4 had the highest score of the upregulated (Fig. S2). Both *SHH* and *TP53* were included in one of the sub-clusters directly involved in the developmental process of embryonic cell lines. Analysis of genes related to “nervous system development and function” (Fig. S1, Table 2) revealed 5 sub-functional clusters involved in the developmental processes. Among these clusters, development of brain, forebrain, neuroepithelial cells, neurons and the central nervous system (CNS) were present (Fig. 4B). Here *TP73*, *AKTIP* and *BMP4* (upregulated) with SHH (downregulated) play a central role with *TP53* acting on CNS and *BAX* on neuron development (Fig. 4B). In the cluster “nervous system development and function”, IPA connected 13 of the differentially expressed genes to each other in a network of different sub-function clusters. The most prominent network was the sub-function cluster “neurogenesis”, which contained *TP53*, *BMP4*, *MEF2C* and *BAX* (upregulated) together with *SOX11*, *GFAP*, *ACHE* and *SHH* (downregulated) (Fig. S2). Also, the “differentiation of neuroglia” and “migration of neurons” harbored 5 genes each.

**Table 1:** 

***A***	
Total number of genes	249 145↑ 104↓
**B**	
Mapped by IPA	120 69↑ 51↓
Not present in IPA	129 76↑ 53↓

A) The number of significantly differentially expressed genes for prp2-MO2 injected 24hpf zebrafish embryos. B) The number of differentially expressed IPA mapped genes (mammalian homologs) and unmapped by IPA (no mammalian homologs found). Arrows represent up- and down-regulated genes respectively.

**Table 2:** List of IPA biological function clusters for differentially expressed genes in 24hpf prp2-MO2 injected zebrafish morphant embryos.

*Category*	*P-value*	*Molecules*
Cellular Development	1,77E-05-9,52E-03	**SHH**, BMP4, CTGF, TP73, AKTIP, SDC4, FOXD3, CD164, **STMN1**, **HBB (includes EG:3043)**, **HMGB1 (includes EG:3146)**, **ASPM**, ANXA1, CRIP2, HNF4A, COL18A1, ECT2, RASGRP2, TP53, **TNC**, PTP4A3, **BHLHB5**, **ACHE**, MMP2, BAX, MLLT1, **TYRP1**, INSM1, **PRKCI**, CD9, ABCC5, MEF2D, IHH, **ALDOA**, MEF2C, NPM1 (includes EG:4869), HMGB3
Developmental Disorder	2,15E-05-7,67E-03	TP53, **SHH**, BMP4, SLC31A1, DKC1, HSPB8, **KAL1**, **ACHE**, MMP2, BAX, **COL1A2**, **PRKCI**, LAMA4, **CRYBA4**, **PHGDH**, ACSL4, IHH
Cell Death	2,39E-05-9,8E-03	**SHH**, CTGF, BMP4, SLC31A1, **CCNI**, TP73, AKTIP, HSPB8, SDC4, **MDH1**, **MYLK**, CCNG1, EGLN1, **STMN1**, **HMGB1 (includes EG:3146)**, ANXA1, ACSL4, IGFBP1, **SNCB**, COL18A1, RASGRP2, TP53, **TNC**, **ACHE**, MMP2, BAX, **PRKCI**, SWAP70, NDEL1, **MCM2**, CD9, ABCC5, MEF2D, **S1PR1**, **ALDOA**, IHH, MEF2C, NPM1 (includes EG:4869), HMGB3
Cellular Assembly and Organization	3,34E-05-8,36E-03	CTGF, CKMT1B, SDC4, **CKB**, **STMN1**, **HMGB1 (includes EG:3146)**, **GFAP**, HNF4A, COL18A1, TP53, **TNC**, **CKM**, PTP4A3, **ACHE**, MMP2, BAX, **PRKCI**, SWAP70, **MCM2**, CD9, LAMA4, H2AFX, **S1PR1**, **GPM6A**, NPM1 (includes EG:4869)
Cardiovascular System Development and Function	4,76E-05-7,67E-03	TP53, **SHH**, CTGF, BMP4, TP73, MMP2, **COL1A2**, EGLN1, LAMA4, **S1PR1**, IHH, MEF2C, COL18A1, ECT2
Gene Expression	5,06E-05-7,67E-03	TP53, **PRKCI, HMGB1 (includes EG:3146)**, TP73, MEF2D, **ATF7IP**, EIF5, MEF2C, BAX, NPM1 (includes EG:4869), HNF4A
Cell Cycle	5,83E-05-9,15E-03	TP53, **SHH**, BMP4, CCNI, TP73, BAX, **CCNG1**, SESN1, **STMN1**, **MCM2**, ANXA1, **S1PR1**, HNF4A, ECT2, NPM1 (includes EG:4869)
Immunological Disease	5,83E-05-8,41E-03	TP53, **SHH**, SWAP70, CD9, **HMGB1 (includes EG:3146)**, TP73, AKTIP, ANXA1, **ACHE**, BAX
Embryonic Development	1,74E-04-7,67E-03	TP53, **SHH**, SLC31A1, BMP4, ABCC5, PTP4A3, IHH, MMP2, BAX, HNF4A, COL18A1
Nervous System Development and Function	1,74E-04-8,23E-03	TP53, **SHH**, BMP4, TP73, AKTIP, **ACHE**, BAX, **STMN1**, EGLN1, CD9, NDEL1, **HMGB1 (includes EG:3146)**, LAMA4, **PHGDH**, **S1PR1**, IHH, COL18A1
Organ Development	1,74E-04-8,36E-03	TP53, **SHH**, BMP4, TP73, AKTIP, MMP2, BAX, FOXD3, **TYRP1**, EGLN1, **HBB (includes EG:3043)**, **S1PR1**, **PHGDH**, IHH, MEF2C, IGFBP1
Neurological Disease	5,74E-04-8,71E-03	**RNF10**, **SHH**, CTGF, **TNNI2**, HSPB8, SDC4, **MDH1**, **EEF1G**, **TUBB2B**, **CKB**, **SPON1**, **STMN1**, **ASPM**, **PHGDH**, LAMB1, ACSL4, **GFAP**, **SNCB**, HNF4A, TP53, **ACHE**, MMP2, BAX, **LRRN1**, SLC14A1, **S1PR1**, PDLIM1, UCK2, NPM1 (includes EG:4869)
Organismal Development	6,42E-04-7,67E-03	TP53, **SHH**, BMP4, SLC31A1, TP73, PTP4A3, **ACHE**, MMP2, BAX, **PRKCI**, **HMGB1 (includes EG:3146)**, SLC14A1, IHH, IGFBP1, COL18A1
Cell Signaling	7,34E-04-4,17E-03	TP53, **CKM**, ANXA1, CKMT1B, BAX, COL18A1

Unbolded molecules are upregulated and bolded molecules are downregulated.

**Figure 5:**
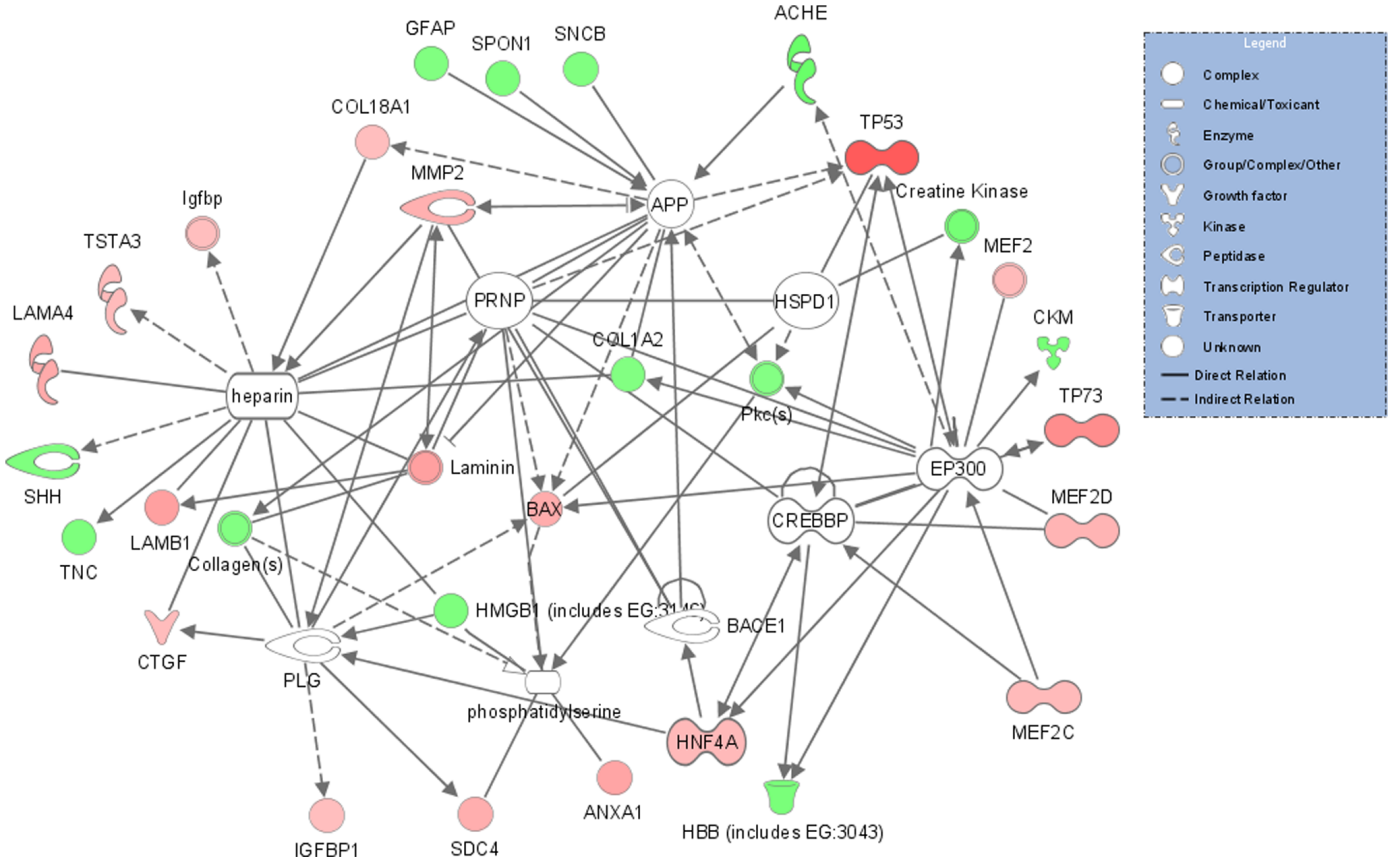
A network for connecting differentially expressed genes. A network for connecting differentially expressed genes from zebrafish 24 hpf prp2-MO2 morphant embryos was identified by IPA in which PRNP, APP, heparin and EP300 occupy a central role. The uncolored protein symbols, including PRNP, are added by IPA to fill network gaps and are not among the analyzed dataset of differentially expressed genes. Red color indicates up- and green downregulation. A significant *prp2* mRNA down-regulation was experimentally demonstrated by qRT-PCR (see Fig. 6).

**Figure 6:**
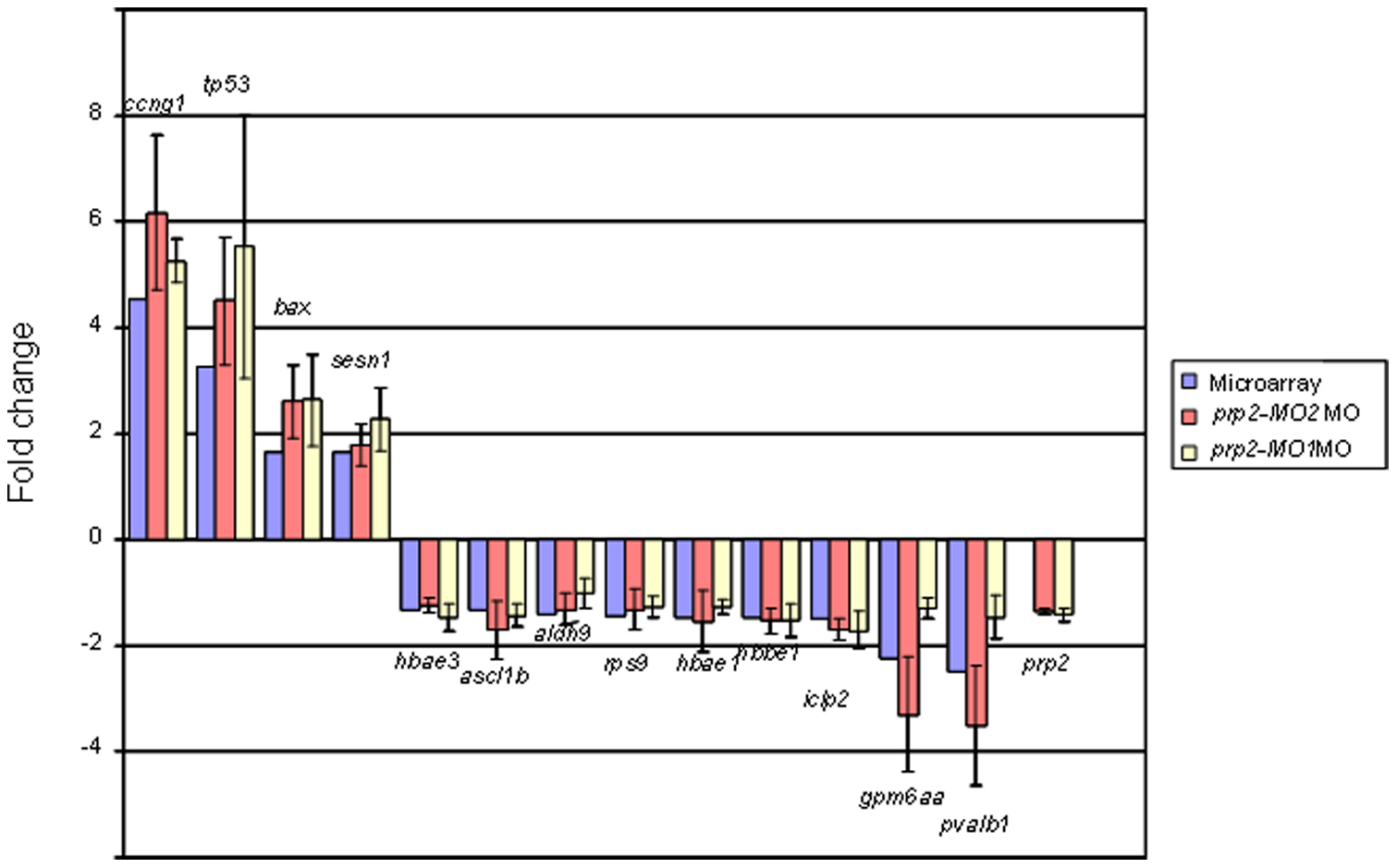
Comparison between microarray data and qRT-PCR data. Microarray data (blue staples), qRT-PCR of RNA from 24 hpf prp2-MO2 injected embryos (red staples) and 24 hpf prp2-MO1 injected embryos (yellow staples) are presented as fold change of expression of 14 genes. Mean fold change (±SD) for qRT-PCR are based on triplicate embryo pools.

In order to explicitly investigate whether the differentially expressed genes in the prp2-MO2 injected embryos could be directly associated with prion gene (*PRNP/prp2*) functions, a customized IPA network with prion and prion associated genes was generated and found to include 18 upregulated and 13 downregulated genes, where the mammalian *prp2* ortholog *PRNP* has a direct relation to *MMP2* and an indirect relation to *BAX* and *TP53* (Fig. 5). However, *PRNP* (*prp2*) itself and some of the other key molecules of the predicted network are not a part of the differentially expressed genes submitted from the microarray dataset (Fig. 5) but showed change in expression by qRT-PCR (Fig. 6; See below and discussion for further clarification).

Validation of the microarray results by qRT-PCR showed a high Pearson's correlation coefficient (0.998) between the microarray and qRT-PCR data (Fig. 6). Further, the corresponding qRT-PCR analysis of the prp2-MO1 morphant transcriptome, using the same 14 gene probe sets, resulted in a comparable pattern of gene expression as the above mentioned results (Pearson's correlation coefficient 0.92). Hence, all of the qRT-PCR expression data strongly support the assumption of a gene specific action by the two *prp2* specific MOs used. In addition, no change in *prp1* gene expression was observed by qRT-PCR analysis of the control and/or the two Prp2 morphants (data not shown).

## Discussion

In the current study, we have targeted *prp2* mRNA translation using two different targeting MOs, prp2-MO1and prp2-MO2. The former had a significantly weaker effect on mortality which may be attributed to its different binding site on the *prp2* mRNA (Fig. 1). A difference in prp2-MO induced mortality compared to what Malaga-Trillo reported may also be explained by different morpholinos [9]. Quantification of gene expression in the morphants prp2-MO1 and prp2-MO2 by qRT-PCR (Fig. 6) implies that the observed changes in gene expression are reliable both with respect to technical quality of the microarray data per se and with respect to gene target specificity of the prp2-MO2 chosen for the microarray experiment. Investigations of the corresponding *prp1* mRNA levels by qRT-PCR showed unaltered levels of this paralogue in the morphants, which suggests that the Prp1 and 2 proteins have gained different main functions in zebrafish. The lethal effect of *prp2* knockdown is in agreement with the proposal of an apoptosis-protective role of the PRNP^C^ protein [11], as also confirmed by hyperdense nuclei observed in DAP1 stained morphants (Fig. 3D). The 24 hpf prp2-MO2 morphant transcriptome (Table S2 and S3) also reveals a cluster of 39 genes involved in cell death, of which 32 were in the sub-cluster of apoptosis (Fig. S1 and 4A). Cyclin G1 (*CCNG1*) the gene with the highest level of upregulated expression in our experiment is known to control *TP53* and *TP73* which are involved in cell cycle control and induction of apoptosis [12], two processes which are augmented in the present study. Furthermore, TP53 and TP73 together with S1PR, BAX and SNCB directly influence apoptosis of cortical neurons, neuroepithelial, neuroblastoma, neural precursor and nervous tissue cells (Fig. 4A). The altered expressions of these genes with their role in apoptosis of neural cells [13], [14] support a putative role for Prp2 in the control of apoptosis of neural cells in zebrafish development. For example, the SNCB protein have been reported to decrease apoptosis of TSM1 neurons [13] and *S1PR*-null mice show a dramatic increase in apoptosis with a decrease in mitosis in the developing nervous system [14].

Both the observed phenotype morphology (Fig. 2B and C) and impaired neurogenesis of the trigeminal ganglion and peripheral neurons (Fig. 3C and D) of the morphants can be correlated to the observed changes in gene expression, clustered to functions associated with embryo development and development of brain, forebrain and central nervous system (Fig. 4B and S2). Two of these key molecules, sonic hedgehog (SHH) and bone morphogenetic protein 4 (BMP4), were both downregulated. SHH acts as a signal molecule in many tissues including the midline structures in the brain, spinal cord and thalamus by the *zona limitans intrathalamica* [15]–[17]. SHH also has a reported ability to act on axonal guidance [18] as well as on neurogenesis where it influences proliferation of neuroblasts and granule cell precursors [19], [20] (Fig. S2). Similar to SHH, BMP4 is involved in many biological processes including negative control of neurogenesis in olfactory epithelial cells and in brain/forebrain development [21]. However, since both SHH and BMP4 are involved in many biological processes, exemplified by their presence in 11 and 9 respectively of the 14 IPA defined biological function clusters of this study (Table 2), further investigations are needed to pinpoint more specific effect-relationships. In addition to *SHH* and *BMP4*, several other genes associated with the processes of neurogenesis, *SOX11*, *GFAP* and *ACHE*, were downregulated in *prp2* knockdown embryos (Fig. S2). SOX11 is suggested to function in the developing nervous system while lack of glial fibrillary acidic protein (*GFAP*) gene function in knock-out mice increases the outgrowth of axons from neurons of the spinal cord [22] (GFAP is central in an IPA cluster related to neurological disorders. See below and Fig. S3). ACHE has been reported to be essential for normal dendrite and axon formation in hippocampal neurons and may function in excitatory synapse development, plasticity and remodeling [22]–[24]. The present microarray and qRT-PCR analyses demonstrate an upregulation of the *BAX* gene, which is a pro-apoptotic protein required for neurotrophin-deprived neuronal apoptosis [25]. Overall, *prp2* knockdown in zebrafish embryos suggests impaired neurogenesis (Fig.'s 2, 3 and 4B) which is in concert with the proposed function of *PRNP* [26], [27]. IPA *PRNP* network analysis was applied to the differentially expressed genes from the prp2-MO2 24 hpf transcriptome and revealed several direct and indirect connections to *PRNP* (Fig. 5). The postulated connection between *PRNP* and the observed upregulation of the pro apoptotic genes *BAX* and *TP53* may indicate an apoptosis protective role of Prp2, as proposed by Aguzzi *et al* [11] and in agreement with the pronounced mortality effect observed in this study. The proposed IPA network includes further nine connections to PRPN, heparin and phosphatidylserine together with 6 genes that were not present in our dataset but together associated with over 30 of the differentially expressed genes (Fig. 5). With the microarray technology used (a 16k library for coverage of 20–25 k genes), it is obvious that not all gene products affected by the MO treatment are included in the presented data set. Next generation sequencing (RNA-seq) will provide a powerful tool which could be used to uncover a magnitude of more details from the complete transcriptome, including all transcripts, alternative splice isoform variants and microRNAs, to allow a deeper understanding of gene networks involved in prion disease.

It is conceivable that the used prp2-MO gene knockdown strategy used here mimics the loss of the normally folded prion protein (PRPN/Prp2), which is part of prion induced pathogenesis. It does however not resemble an increase in misfolded prion protein and its aggregation into high molecular weight fibers which characterize prion disease. In a study of sporadic Creutzfelt-Jakob disease (sCJD), 79 upregulated and 275 downregulated genes were identified [28]. The major alterations in sCJD brain samples included upregulation of the genes encoding immune and stress-response factors and elements involved in cell death and cell cycle, as well as prominent downregulation of genes encoding synaptic proteins. There was no obvious common pattern of differentially expressed genes between the respective clusters or gene lists and thus providing no evidence for that the loss of the normally folded prion protein function would be a major cause of prion induced pathogenesis [28]. Of 230 genes in the sCJD gene list, 4 are however common with the Prp2 morphants: *GFAP*, phosphoglycerate dehydrogenase (*PHGDH*), SWAP-70 protein (*SWAP70*) and *TP53*. *SWAP70*, *PHGDH* and *TP53* are included in the IPA apoptosis cluster (Fig. 4A and B). When extending the comparisons between differentially expressed genes to include two mouse scrapie models [29], [30] only 1 gene (*GFAP*) is in common for all 4 studies and two when comparing the 3 mammalian (*GFAP*, *ABCA1*). *GFAP* is proposed by IPA to be indirectly linked to PRNP and neurogenesis (Fig. 5; S2). Comparing the prp2 morphant with the mouse scrapie model [29] revealed 5 genes in common: *GFAP*, CD9 antigen (*CD9*), solute carrier family 14 member 1 (*SLC14A1*), heat shock 22kDa protein 8 (*HSPB8*) and syndecan 4 (*SDC4*). An IPA cluster analysis could link all the 5 genes to neurological disorders (Fig. S3). *CD9* and *HSPB8* included in the IPA apoptosis cluster (Fig. 4A). When comparing each mouse study alone against the sCJD data [29], [30] the overlapping gene numbers are 11–14 which is not far from the 4–5 for the zebrafish-sCJD or zebrafish-mouse, taken into consideration the fact that only 120 of 249 of the zebrafish genes have known mammalian homologs (Table 1). With this background it is fair to state that the zebrafish prp2 morphants provide a good model to shed light on the normal biological function of the prion protein. Our gene expression study is based on depleting the mammalian prion protein ortholog from the developing zebrafish embryo and has been able to link the zebrafish *prp2* gene to biological processes including cell death, embryo development and neurogenesis. Although not many genes overlap when comparing 3 mammalian scrapie related transcriptomes with the prp2 gene knockdown zebrafish transcriptome, it is interesting to see one gene which is linked to a variety of neurological disorders in common for all 4 studies (Fig. S3). Many genes associated with these processes have been uncovered but still a more distinct mechanism of action for Prp2 remains to be found. We conclude that the data from this study, together with the paper of Malaga-Trillo *et al.* [9], place zebrafish as a highly relevant model to address the still largely unanswered questions of the molecular mechanisms underlying prion protein function, aggregation and the corresponding disease pathology.

## Materials and Methods

### Zebrafish and prp2-MO microinjections

Newly fertilized embryos were obtained from zebrafish reared in Alestrom Zebrafish Lab http://zebrafish.no/ (http://zebrafish.no) according to husbandry standards as described in Alestrom Lab Standard Operating Procedures. As an AAALAC (http://www.aaalac.org) accredited laboratory, all activities in the unit are subject for evaluation by an IACUC. In all of the experiments presented, only zebrafish embryos of age up to 24 hpf were used. For titration of dose response, two MOs specific for the 5′UTR of GenBank sequence AJ620614.1 (Fig. 1) were injected in single cell embryos at the following concentrations: 0.1, 0.15, 0.5 and 1mM. The mortality rate for negative control embryos (non-injected and mock injected with 1× Danieu solution) was below 20% (data not shown).

### Method for immobilizing embryos and microscopy

Prp2 morphants larvae were embedded in CyGEL Sustain™ (Biostatus Limited, Leicestershire, UK) and investigated for phenotype using a Nikon AZ 100 fitted with Nikon Digital Sight DS Ri1 camera. Morphants and wild type embryo were fixed in 4% PFA and transferred to methanol for immunofluorescence.

### Whole mount immunofluorescence

Wild type and morphant embryos were dechorinated and fixed in 4% PFA and then dehydrated in 70% methanol. Fluorescent immunohistochemistry was carried out on rehydrated and permeabilized embryos (10 min 1% Triton X-100, Sigma) after a 2 hr blocking in 5% dry milk dissolved in 1× Phosphate Buffered Saline Tween 20 (PBST; 1× PBS, 0.1% Tween). Antibodies applied included the HNK-1 specific Zn12 [31] (Developmental studies Hybridoma Bank, IA, USA) and a rabbit serum against Prp2 [32]. The former was used at a 200 fold dilution in 2% dry milk with 1% DMSO in 1× PBST, whereas the latter was diluted 100×. After over night incubation at 4°C with the primary antibodies, the embryos were washed thoroughly in 1× PBST and incubated with Alexa conjugated secondary antibodies diluted 200× (Invitrogen, Ca, USA) for 2 hours. Final 1× PBST washes was carried out before glycerol mounting and microscopy. All images were captured using a Zeiss Axioplan Z1 fitted with the Apotome system for structured illumination microscopy. The images were post processed deconvolved and visualized in 3D using Zeiss Axiovison software.

### RNA isolation

Total RNA from injected control (1× Danieau solution) and MO injected 24 hpf zebrafish embryo was isolated for microarray and qRT-PCR analysis. Briefly, pools of 50 embryos were treated with 1 mL of Trizol reagent (Invitrogen, CA, U.S.A). Homogenization was carried out using MagNA Lyser Beads (Roche Diagnostics Gmbh, Mannheim, Germany) and total RNA isolation was performed according to the TRIzol manufacturer (Invitrogen), followed by a 15 min DNaseI treatment (Qiagen Hilden, Germany) at 25°C. Further RNA purification was conducted using RNAeasy mini kit (Qiagen). After purification, the samples were eluted in 50 µL of RNase-free water and aliquoted for microarray and qRT-PCR analyses. RNA yield and integrity were determined using a NanoDrop ND-1000 instrument (NanoDrop Technologies, DE, U.S.A) and Agilent 2100 bioanalyzer (Agilent Technologies, CA, U.S.A.) respectively. None of the samples showed signs of degradation or impurities (260/280 and 260/230 >1.8, RIN >8.0).

### Microarray strategy

The total RNA from zebrafish embryo morphant pools was compared to total RNA from Danieau-injected control pools. The replicate embryo pools were analyzed in two sets of dye-swaps making a total of four hybridizations. Normalized microarray datasets did not result in significantly altered gene expression for zebrafish housekeeping gene *β*-actin2 (172 probes in our arrays). Like other microarray gene expression profiling studies, we see a reproducible high correlation between microarray and qPCR data [33].

### Data deposition

Microarray data have been submitted to the European Bioinformatics Institutes Array Express accessible through experiment accession number E-MEXP-2365.

### Linear RNA amplification, target labeling and hybridization

One µg total RNA was linearly amplified and labeled, using an Amino Allyl MessageAmp™ II aRNA amplification kit (Ambion TX, U.S.A). Five µg of the resulting amplified RNA (aRNA) from the MO-treated and control groups were labeled either with Cy3-dUTP or Cy5-dUTP (GE Healthcare CT, U.S.A). The labeled targets were examined for amplification yield and incorporation efficiency by measuring the aRNA concentration at 260 nm, Cy3 incorporation at 550 nm and Cy5 incorporation at 650 nm using a Nanodrop ND-1000. One to 5 µg of each labeled target aRNA were mixed with 9 µL of 25× fragmentation buffer (Agilent Technologies) and the final volume adjusted to 225 µL with RNAse free H_2_O followed by incubation for 30 min at 60°C. The hybridization solution was prepared by adding 220.5 µL of 2× hybridization buffer (Agilent) and 4.5 µL (10 µg/µL) sonicated herring sperm DNA (Promega WI, U.S.A) to the labeled target aRNA. Microarray slides were pre-hybridized at 42°C, 60 min using 0.1% Bovine Serum Albumin Fraction V, 5× SSC and 0.1% SDS. Hybridization was performed at 60°C for 16 hours using Agilent gasket slides G2534-60003, hybridization chamber and oven according to the manufacturer (Agilent Technologies). Microarray slides were then washed 3× 5 min in 0.5× SSC and 0.01% SDS with the first wash at 42°C and the next two at room temperature. Finally, slides were washed 3 times at room temperature with 0.06× SSC and dried immediately by centrifuging at 1000 rpm for 1 minute.

### Zebrafish oligonucleotide library and microarray construction

The Zebrafish OligoLibrary™ (Compugen, Ontario, Canada) used was composed of 16,399 (16k) 65-mer oligonucleotide probes, originally representing 16,228 clusters predicted to be representing unique genes plus 171 positive control probes. All probe sequences were selected from the 3′ UTR in order to cover a maximum number of splice variants. The oligonucleotides were printed on CMT UltraGAPS slides at the Norwegian Microarray Consortium (NMC, www.microarray.no ) with a Microgrid II (BioRobotics, Oslo). Probe annotation and information about Unigene ID, Unigene description, Gene symbol, GO ontology and human and mouse homologue ID and descriptions were obtained with the Unigene build release, using the Genome institute of Singapore Unigene & Gene Ontology Annotation Tool Genome institute of Singapore (http://giscompute.gis.a-star.edu.sg/~govind/unigene_db/). GenBank accession numbers were used as a query. In the present study annotations were made using *Danio rerio* Build #107-58.

### Pathway analysis and functional profiling

For pathway and biological function analysis of significantly differentially expressed genes, the Ingenuity Pathway Analysis (IPA) program was used. The results were analyzed using the Unigene & Gene Ontology Annotation Tool for identification of mammalian (human, mouse and rat) orthologs before IPA software analysis. The output results comprise gene networks, pathways and functional clusters, where given scores and p-values are based on numbers of uploaded genes in the cluster or network and the size of cluster or network in the IPA knowledge database. Fisher's exact test was used to determine the statistical significance and the likelihood of random clustering to a pathway. Scores of 2 or higher have at least a 99% likelihood of not being generated by chance alone. Mammalian orthologs from IPA are displayed using human homolog identifiers whereas zebrafish genes and proteins are annotated according to standard zebrafish nomenclature guidelines (Table S1).

### Quantitative real-time PCR

The accuracy of the microarray results were validated by analyzing 14 differentially expressed genes by qRT-PCR using the same samples of total RNA. The intron spanning qRT-PCR primers were designed using “Universal Probe Library Assay Design Center/ProbeFinder version 2.40” ((http://www.roche-applied-science.com/sis/rtpcr/upl/adc.jsp). Primer sequences are summarized in Table 3. Template cDNAs were produced from 1 µg total RNA using Superscript III Reverse Transcriptase according to manufacturer (Invitrogen). qRT-PCR analysis was carried out on a LightCycler 2.0 instrument using LightCycler®FastStart DNA MasterPLUS SYBR Green I (Roche). All reactions were performed in triplicate with 0.5 µL of the respective primers (10 pmol) and 2.0 µL enzyme mix in a total reaction volume of 10 µL. The PCR conditions were: 95°C for 10min followed by 40 cycles of 10 s at 95°C, 10 s at 60°C and 6 s at 72°C. Melting curve analysis and agarose gel electrophoresis was carried out to confirm PCR products. Data analysis with crossing point (Cp) was determined by use of the maximum-second-derivative function in the LightCycler®Software version 4.0 (Roche). Relative gene expression levels were obtained using beta-actin as the internal reference gene and efficiency correction with external standards. The mean gene expression values for each group (exposed versus control) were compared and p-values calculated using unpaired Student's *t*-test.

**Table 3:** qRT-PCR primers (5′-3′) used to validate microarray and β-actin.

*GenBank*	*Symbol*	*Unigene Descp*	*Forward Primer*	*Reverse Primer*
AI959372	*ccng1*	Cyclin G1	attgaggatcagcacgagtatg	aaccaggtctccagctttaaca
U60804	*Tp53*	Tumor protein p53	ctgaagtggtccgcagatg	ttgccctccactcttatcaaa
BI891654	*bax*	Bcl2-associated X protein	ccgtgagatcttctctgatgg	gtcaggaaccctggttgaaa
BI878036	*sesn1*	Sestrin 1	gctgctcaccaaagaacaca	cacagcatggatcagctcag
AI353083	*hbae3*	Hemoglobin alpha embryonic-3	atcggccgtgagactcttt	ggagagttggggcttaggtc
U14590	*ascl1b*	Achaete-scute complex-like 1b (Drosophila)	ccgatgaaggaagctacgag	caatctttagtagccattgaagcac
BG302998	*aldh9*	Similar to Aldehyde dehydrogenase 9 family, member A1 like 1	ataacacaacctttggcttgg	gttcgcagcaactctgtgg
AI964216	*rps9*	Ribosomal protein S9	ggaggttgcagactcaggtc	gggatgttcacaacctgctta
BE693186	*hbae1*	Hemoglobin alpha embryonic-1	aaagtcatccttccacaatgagt	ggggtagacaatcaacatcctg
AF082662	*hbbe1*	Hemoglobin beta embryonic-1	ctccacgtagatcctgacaactt	tgaacttcaggtgtgaatccag
AF116539	*iclp2*	Invariant chain-like protein 2	agtgttgggcccttaattcc	ttcactcgccatttttcctc
BI840762	*gpm6aa*	Similar to Glycoprotein M6A	aatcacaggacaaggcaaca	tcagacagcactccatacatcc
AF180888	*pvalb2*	Parvalbumin 2	gacaacagcggcttcattg	actctagcgttggccttgaa
AY438684.1	*prp2*	Prion protein 2	caatcgcccaagagaggac	caagagccagacagagcaatag
AY438683.1	*prp1*	Prion protein 1	cgcttcttcaacctttttatgg	gcctttctttcctcctgatgt
AF025305.1	*β-actin*	β-actin	aaggccaacagggaaaagat	gtggtacgaccagaggcatac

### Data analysis and statistical methods

Microarray slides were scanned using a GenePix 4000B (Axon instrument, Foster City, CA, U.S.A) with threshold level just before saturation of several spots. Raw data generated from GenePix were imported into the Bioconductor package LIMMA and corrected for background. For within array and between array normalization, print tip Loess and scale was used. An empirical Bayes moderated t-test [34]–[37] was applied to detect differentially expressed genes across treated and control samples. P-values were corrected for multiple testing using Benjamini–Hochberg (BH) method [38] and p-values<0.05 were selected as differentially expressed genes. The generated gene list was further filtered for genes with low intensity and with small changes in expression. In the averaged normalized MA-Plot, the majority of genes were clustered between M-values of ±0.4 (fold change ±1.3) and selected to the threshold criteria for the differentially expressed gene list. Another threshold criterion was A-values<6, representing empty or weak intensity spots, which were excluded from the final gene list. Relative gene expression values (qRT-PCR) for each target gene were expressed as mean fold change obtained from 3 replicate samples. Correlation between microarray and qRT-PCR data for 14 genes was analyzed using Pearson product-moment correlation coefficient.

## Supporting Information

Figure S1 IPA generated functional clusters of differentially expressed genes. IPA generated functional clusters of differentially expressed genes in 24 hpf zebrafish prp2 morphant (prp2-MO2) embryos. All clusters have p-values<0.05 and are ranked by decreasing p-values. Corresponding p-values are illustrated in Table 3. The numbers in the pie diagram corresponds to the numbers of genes with significantly different expression in each cluster. The clusters appear in the same order in the diagram as in the ID pane.
(0.24 MB TIF)

Figure S2 IPA cluster analyses of significant differentially expressed genes. IPA cluster analyses of significant differentially expressed genes for 24 hpf zebrafish Prp2 morphant embryos. The cluster reveals genes involved in nervous system function. Red color indicates up- and green downregulation.
(2.60 MB TIF)

Figure S3 Comparative analysis of differentially expressed genes. Comparative analysis of differentially expressed genes between the prp2-MO2 morphant and a mouse scrapie model [29] reveals 5 common genes: GFAP, CD9, HSPB8, SDC4 and SLC14A1. The functional clustering by IPA indicates a central role for these genes in neurological disorders: Huntington disease, Parkinson disease, retinopathy, Alexander disease, motor neuron disease, hydrocephalus and hypertrophy of astrocytes.
(0.42 MB TIF)

Table S1 Nomenclature and relationship between prion protein genes and proteins. The arguments for prp2/Prp2 being the mammalian PRNP/PRP homolog is summarized. To simplify reading we have used the old conventional names (in bold) in the article and in Table S1.
(0.04 MB DOC)

Table S2 Fold change of differentially expressed genes mapped by IPA in 24 hpf prp2-MO2 MO-injected zebrafish embryos.
(0.15 MB DOC)

Table S3 Fold change of differentially expressed genes which were not mapped by IPA in 24 hpf prp2-MO2 injected zebrafish embryos. These genes are annotated with zebrafish gene symbols obtained from the Genome Institute in Singapore.
(0.15 MB DOC)
