# Supplementary figures and images for: Early Embryonic Gene Expression Profiling of Zebrafish Prion Protein (Prp2) Morphants
Source: PLoS One. 2010 Oct 22;5(10):e13573. doi: 10.1371/journal.pone.0013573 (PMC2962645; doi:10.1371/journal.pone.0013573)

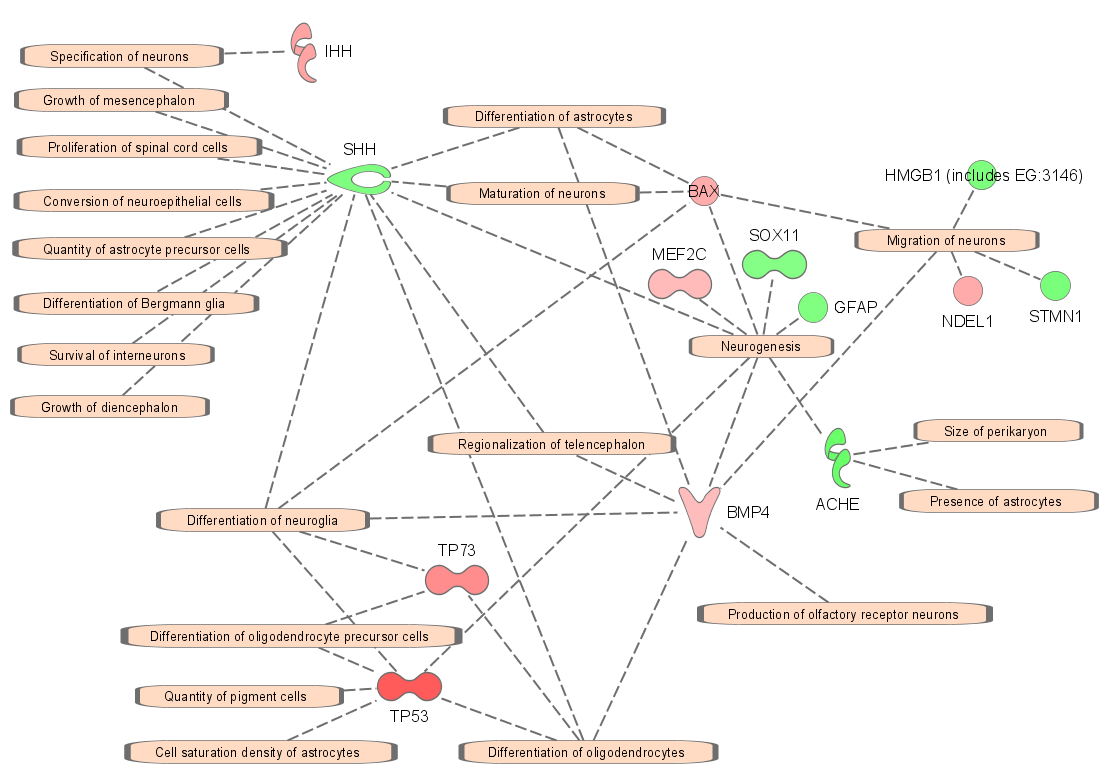

Supplement: Figure S2 — IPA cluster analyses of significant differentially expressed genes. IPA cluster analyses of significant differentially expressed genes for 24 hpf zebrafish Prp2 morphant embryos. The cluster reveals genes involved in nervous system function. Red color indicates up- and green downregulation. (2.60 MB TIF) [file pone.0013573.s002.tif]
