# Supplementary material for: Early Embryonic Gene Expression Profiling of Zebrafish Prion Protein (Prp2) Morphants
Source: PLoS One. 2010 Oct 22;5(10):e13573. doi: 10.1371/journal.pone.0013573 (PMC2962645; doi:10.1371/journal.pone.0013573)
Supplement: Table S1 — Nomenclature and relationship between prion protein genes and proteins. The arguments for prp2/Prp2 being the mammalian PRNP/PRP homolog is summarized. To simplify reading we have used the old conventional names (in bold) in the article and in Table S1. (0.04 MB DOC) [file pone.0013573.s004.doc]

**Table S1**. Nomenclature and relationship between prion protein genes and proteins. The arguments for *prp2*/Prp2 being the mammalian *PRNP*/PRP homolog is summarized. To simplify reading we have used the old conventional names (in bold) in the article and in Table S1.

| Zebrafish prion protein genes, present (left) and previously (right) nomenclature^1^. | | Molecular characterization and developmental expression.  (The protein nomenclature for zebrafish prion proteins used is Prp1, Prp2 and Prp3, which according to the present formally correct zebrafish nomenclature should be Prnprs1, Prnprs3 and Prnp respectively). |
| --- | --- | --- |
| *prnprs1*  ***prp1*** | *stPrP-1*  ***PrP1***  *zgc:123004*  *PrP-1* | Alignment of the conserved sequence motifs of the alpha-helical C-terminal domain between zebrafish Prp1 and human PRP showed 25% amino acid identities^2^.  prp1 presented a highly spatially restricted expression in the central and peripheral nervous systems^2^ . |
| *prnprs3*  ***prp2*** | *ZePrP-3* ***PrP2***  *PrP-2 zgc:123003 stPrP-3* | Alignment of the conserved sequence motifs of the alpha-helical C-terminal domain between zebrafish Prp2 and human PRP resulted in 33% amino acid identity^2^.  prp2 transcripts were found widely distributed within the CNS and a high expression of zebrafish prp2 has been demonstrated in the brain, kidney and posterior intestine^2^.    Differential developmental expression patterns observed among *prp1*, *prp2*, and *prp3* are much in favor of a functional link between *prp2* and tetrapod prion genes due to a widespread localization of both transcripts in the CNS and in peripheral organs^2^.  The *PRNP* gene is present in all tetrapods, and its homologue in fish is  *prp2* ^3,4,5^. |
| *prnp*  ***prp3*** | ***PrP3***  *PrP-rel-2*  *PrP-like* | Alignment of the conserved sequence motifs between fish proteins similar to tetrapod *PRP*s demonstrated that the fish duplicated prion gene long forms, *prp1 and prp2*, are more structurally related to human *PRNP* than fish *prp3* or *sho* sequences^2^.  Zebrafish prp3 expression profile lacks one of the characteristic key features of tetrapod PrPs, i.e. intense expression in the CNS. No prp3 signal could be detected by whole-mount in situ hybridization in kidney, liver, and posterior intestine during embryonic and larval development^2^. |

Genes/Markers/Clones data for this paper were retrieved from the Zebrafish Information Network (ZFIN), University of Oregon, Eugene, OR 97403-5274; World Wide Web URL: [http://zfin.org](http://zfin.org/)/; September 10, 2010.

Sprague, J., Bayraktaroglu, L., Clements, D., Conlin, T., Fashena, D., Frazer, K., Haendel, M., Howe, D.G., Mani, P., Ramachandran, S., Schaper, K., Segerdell, E., Song, P., Sprunger, B., Taylor, S., Van Slyke, C.E., and Westerfield, M. (2006). The 22 Zebrafish Information Network: the zebrafish model organism database. Nucleic Acids Res. 34: D581-D585.

^2^ Cotto E, Andre M, Forgue J, Fleury HJ, Babin PJ (2005) Molecular characterization, phylogenetic relationships, and developmental expression patterns of prion genes in zebrafish (Danio rerio). FEBS J, 272: 500-513

^3^ Rivera-Milla E, Oidtmann B, Panagiotidis CH, Baier M, Sklaviadis T, Hoffmann R et al.: Disparate evolution

of prion protein domains and the distinct origin of Doppel- and prion-related loci revealed by fish-to-mammal

comparisons (2006)FASEB J, 20: 317-319.

^4^ Premzl Marko, Gready JE, Jermiin LS, Simonic T, and Graves JAM (2004) Evolution of Vertebrate Genes Related to Prion and Shadoo Proteins—Clues from Comparative Genomic Analysis. Mol. Biol. Evol. 21(12):2210–2231.

^5^ Premzl M and Gamulin V (2007) Comparative genomic analysis of prion genes. BMC Genomics 2007, 8:1
