# Supplementary material for: Early Embryonic Gene Expression Profiling of Zebrafish Prion Protein (Prp2) Morphants
Source: PLoS One. 2010 Oct 22;5(10):e13573. doi: 10.1371/journal.pone.0013573 (PMC2962645; doi:10.1371/journal.pone.0013573)
Supplement: Table S2 — Fold change of differentially expressed genes mapped by IPA in 24 hpf prp2-MO2 MO-injected zebrafish embryos. (0.15 MB DOC) [file pone.0013573.s005.doc]

Table S2: Fold change of differentially expressed genes mapped by IPA in 24 hpf prp2-MO2 MO-injected zebrafish embryos.

| *GenBank* | *Zebrafish symbols* | *Mammalian symbols* | *Mammalian homolog description* | *Fold change* |
| --- | --- | --- | --- | --- |
| AI959372 | *ccng1* | CCNG1 | cyclin G1 | 4,2 |
| U60804 | *tp53* | TP53 | tumor protein p53 | 3,36 |
| BI430331 | *LOC796249* | ABCC5 | ATP-binding cassette, sub-family C (CFTR/MRP), member 5 | 2,74 |
| AY233269 | *tp73* | TP73 | tumor protein p73 | 2,33 |
| BI850028 | *lamb1* | LAMB1 | laminin, beta 1 | 1,94 |
| Y08426 | *ihhb* | IHH | Indian hedgehog homolog (Drosophila) | 1,88 |
| AW128428 | *anxa1a* | ANXA1 | annexin A1 | 1,84 |
| AW175429 | *LOC564961* | DNTTIP2 | deoxynucleotidyltransferase, terminal, interacting protein 2 | 1,75 |
| BG728552 | *im:7138247* | LAMA4 | laminin, alpha 4 | 1,75 |
| BI563231 | *zgc:65861* | H1F0 | H1 histone family, member 0 | 1,73 |
| AW154176 | *LOC555064* | NDEL1 | nudE nuclear distribution gene E homolog (A. nidulans)-like 1 | 1,72 |
| BI704240 |  | TCAG7.1177 | opposite strand transcription unit to STAG3 | 1,72 |
| BI891654 | *bax* | BAX | BCL2-associated X protein | 1,7 |
| BI878036 | *sesn1* | SESN1 | sestrin 1 | 1,69 |
| AI959694 | *sdc4l* | SDC4 | syndecan 4 | 1,65 |
| BM181739 | *LOC793660* | OC90 | otoconin 90 | 1,64 |
| BM182277 | *mmp2* | MMP2 | matrix metallopeptidase 2 (gelatinase A, 72kDa gelatinase, 72kDa type IV collagenase) | 1,61 |
| BG305891 | *crip2* | CRIP2 | cysteine-rich protein 2 | 1,6 |
| AI878489 | *zgc:64202* | HSPB8 | heat shock 22kDa protein 8 | 1,58 |
| AA495004 | *zgc:113194* | ACSS1 | acyl-CoA synthetase short-chain family member 1 | 1,57 |
| BG304092 | *mef2d* | MEF2D | myocyte enhancer factor 2D | 1,53 |
| BI892229 | *cd9l* | CD9 | CD9 molecule | 1,52 |
| BI880152 | *zgc:112073* | HMGB3 | high-mobility group box 3 | 1,52 |
| AI544884 | *upb1* | UPB1 | ureidopropionase, beta | 1,52 |
| BI885972 | *LOC797658* | RASGRP2 | RAS guanyl releasing protein 2 (calcium and DAG-regulated) | 1,5 |
| BI888958 | *krt8* | KRT7 | keratin 7 | 1,49 |
| BM182319 | *slc31a1* | SLC31A1 | solute carrier family 31 (copper transporters), member 1 | 1,49 |
| AI667665 | *aktip* | AKTIP | AKT interacting protein | 1,47 |
| BI890279 | *LOC100007704* | SLC7A8 | solute carrier family 7 (cationic amino acid transporter, y+ system), member 8 | 1,47 |
| BI982003 | *smyd2* | SMYD2 | SET and MYND domain containing 2 | 1,46 |
| AI721419 | *acsl4* | ACSL4 | acyl-CoA synthetase long-chain family member 4 | 1,45 |
| BM024762 | *h2afx* | H2AFX | H2A histone family, member X | 1,45 |
| BG883304 | *hnf4a* | HNF4A | hepatocyte nuclear factor 4, alpha | 1,43 |
| BM095862 | *arrdc2* | ARRDC2 | arrestin domain containing 2 | 1,42 |
| AW232057 | *cd164* | CD164 | CD164 molecule, sialomucin | 1,41 |
| BI878159 | *ect2* | ECT2 | epithelial cell transforming sequence 2 oncogene | 1,41 |
| AW165272 | *zgc:66241* | ESF1 | ESF1, nucleolar pre-rRNA processing protein, homolog (S. cerevisiae) | 1,41 |
| U66569 | *mef2ca* | MEF2C | myocyte enhancer factor 2C | 1,41 |
| AW420304 | *zgc:136639* | MLLT1 | myeloid/lymphoid or mixed-lineage leukemia (trithorax homolog, Drosophila); translocated to, 1 | 1,41 |
| AI545016 | *wu:fb73f07* | NENF | neuron derived neurotrophic factor | 1,41 |
| AW165069 | *zgc:136632* | SLC14A1 | solute carrier family 14 (urea transporter), member 1 (Kidd blood group) | 1,4 |
| AW019458 | *zgc:85843* | TM6SF1 | transmembrane 6 superfamily member 1 | 1,4 |
| AI964264 | *ckmt1* | CKMT1B | creatine kinase, mitochondrial 1B | 1,39 |
| BG303575 | *eif5* | EIF5 | eukaryotic translation initiation factor 5 | 1,39 |
| BM095161 | *ptp4a3* | PTP4A3 | protein tyrosine phosphatase type IVA, member 3 | 1,39 |
| BM083940 | *zgc:100864* | TSTA3 | tissue specific transplantation antigen P35B | 1,39 |
| BI888721 | *npm1* | NPM1 (includes EG:4869) | nucleophosmin (nucleolar phosphoprotein B23, numatrin) | 1,38 |
| BI877751 | *pdlim1* | PDLIM1 | PDZ and LIM domain 1 (elfin) | 1,38 |
| NM_131342 | *bmp4* | BMP4 | bone morphogenetic protein 4 | 1,37 |
| BE693178 | *ctgf* | CTGF | connective tissue growth factor | 1,36 |
| AF052249 | *foxd3* | FOXD3 | forkhead box D3 | 1,36 |
| BI880684 | *rcan3* | RCAN3 | RCAN family member 3 | 1,36 |
| AI793391 | *LOC555313* | SWAP70 | SWAP-70 protein | 1,36 |
| AF295407 | *adh8a* | ADH6 | alcohol dehydrogenase 6 (class V) | 1,35 |
| BG303234 | *zgc:92314* | EGLN1 | egl nine homolog 1 (C. elegans) | 1,35 |
| BI885558 | *slc4a2* | SLC4A3 | solute carrier family 4, anion exchanger, member 3 | 1,35 |
| BI867572 | *zgc:162874* | TEX2 | testis expressed 2 | 1,35 |
| AW116780 | *cacng1* | CACNG1 | calcium channel, voltage-dependent, gamma subunit 1 | 1,34 |
| AI794059 | *cdh6* | CDH6 | cadherin 6, type 2, K-cadherin (fetal kidney) | 1,34 |
| BI867554 | *zgc:86604* | BXDC5 | brix domain containing 5 | 1,33 |
| AW154707 | *cdv3* | CDV3 | CDV3 homolog (mouse) | 1,33 |
| AJ299409 | *igfbp1* | IGFBP1 | insulin-like growth factor binding protein 1 | 1,33 |
| BI877634 | *LOC796258* | UCK2 | uridine-cytidine kinase 2 | 1,33 |
| AI721634 | *cad* | CAD | carbamoyl-phosphate synthetase 2, aspartate transcarbamylase, and dihydroorotase | 1,32 |
| BM025541 | *col18a1* | COL18A1 | collagen, type XVIII, alpha 1 | 1,32 |
| BI886184 | *dkc1* | DKC1 | dyskeratosis congenita 1, dyskerin | 1,32 |
| BI886057 | *flj12949l* | KRI1 | KRI1 homolog (S. cerevisiae) | 1,32 |
| AW777591 | *polr1a* | POLR1A | polymerase (RNA) I polypeptide A, 194kDa | 1,32 |
| AW778181 | *aspm* | ASPM | asp (abnormal spindle) homolog, microcephaly associated (Drosophila) | -1,32 |
| BI840896 | *bhlhb5* | BHLHB5 | basic helix-loop-helix domain containing, class B, 5 | -1,32 |
| NM_173222 | *ckb* | CKB | creatine kinase, brain | -1,32 |
| BM071225 | *aldoab* | ALDOA | aldolase A, fructose-bisphosphate | -1,33 |
| AI354172 | *eef1g* | EEF1G | eukaryotic translation elongation factor 1 gamma | -1,33 |
| AW128561 | *nutf2* | RNF10 | ring finger protein 10 | -1,33 |
| U85091 | *sox11b* | SOX11 | SRY (sex determining region Y)-box 11 | -1,33 |
| AF165216 | *tmod4* | TMOD4 | tropomodulin 4 (muscle) | -1,33 |
| BI885931 | *atf7ip* | ATF7IP | activating transcription factor 7 interacting protein | -1,34 |
| BE201627 | *zmlck1* | MYLK | myosin light chain kinase | -1,34 |
| AW115626 | *mcm2* | MCM2 | minichromosome maintenance complex component 2 | -1,35 |
| AF390109 | *prkci* | PRKCI | protein kinase C, iota | -1,35 |
| AJ318213 | *col1a2* | COL1A2 | collagen, type I, alpha 2 | -1,37 |
| AW058863 | *si:ch211-132p20.4* | SLC38A2 | solute carrier family 38, member 2 | -1,37 |
| AW777320 | *gfap* | GFAP | glial fibrillary acidic protein | -1,39 |
| AB026980 | *odz4* | ODZ4 | odz, odd Oz/ten-m homolog 4 (Drosophila) | -1,4 |
| AW232264 | *LOC791988* | SNCB | synuclein, beta | -1,4 |
| AI444198 | *zgc:92040* | PRODH2 | proline dehydrogenase (oxidase) 2 | -1,41 |
| U30710 | *shhb* | SHH | sonic hedgehog homolog (Drosophila) | -1,41 |
| BM185207 | *glud1b* | GLUD2 | glutamate dehydrogenase 2 | -1,42 |
| BI982166 | *MGC165618* | HMGB1 (includes EG:3146) | high-mobility group box 1 | -1,42 |
| U14940 | *tnc* | TNC | tenascin C (hexabrachion) | -1,43 |
| BG302998 | *-* | ALDH9A1 | aldehyde dehydrogenase 9 family, member A1 | -1,44 |
| BI882594 | *aqp3* | AQP3 | aquaporin 3 (Gill blood group) | -1,44 |
| BI839973 | *zgc:154087* | DHRS7 (includes EG:51635) | dehydrogenase/reductase (SDR family) member 7 | -1,44 |
| AF163310 | *kal1a* | KAL1 | Kallmann syndrome 1 sequence | -1,44 |
| AL718237 | *MGC158377* | MCM6 | minichromosome maintenance complex component 6 | -1,44 |
| AB006087 | *spon1b* | SPON1 | spondin 1, extracellular matrix protein | -1,44 |
| AF402599 | *lyz* | LYZL2 | lysozyme-like 2 | -1,45 |
| AF082662 | *hbbe1* | HBB (includes EG:3043) | hemoglobin, beta | -1,46 |
| H56788 | *zgc:109979* | CRYBA4 | crystallin, beta A4 | -1,48 |
| BI704376 | *mdh1a* | MDH1 | malate dehydrogenase 1, NAD (soluble) | -1,48 |
| BI428507 | *zgc:110159* | STMN1 | stathmin 1/oncoprotein 18 | -1,48 |
| AI626492 | *tubb5* | TUBB2B | tubulin, beta 2B | -1,48 |
| AI626799 | *insm1b* | INSM1 | insulinoma-associated 1 | -1,49 |
| AI544976 | *mfsd2* | MFSD2 | major facilitator superfamily domain containing 2 | -1,5 |
| BM104016 | *zgc:64204* | CKM | creatine kinase, muscle | -1,51 |
| BE017206 | *zgc:158424* | CCNI | cyclin I | -1,55 |
| AI883922 | *zgc:65956* | PHGDH | phosphoglycerate dehydrogenase | -1,56 |
| BM081047 | *zgc:136591* | RPL22L1 | ribosomal protein L22-like 1 | -1,56 |
| AI958208 | *LOC794322* | HNRNPUL1 | heterogeneous nuclear ribonucleoprotein U-like 1 | -1,62 |
| BI882649 | *tyrp1b* | TYRP1 | tyrosinase-related protein 1 | -1,64 |
| BC076071 | *ache* | ACHE | acetylcholinesterase (Yt blood group) | -1,65 |
| AI522688 | *zgc:154059* | TAT | tyrosine aminotransferase | -1,71 |
| BM155941 | *tnni2* | TNNI2 | troponin I type 2 (skeletal, fast) | -1,71 |
| BE558113 | *LOC569755* | COL6A1 | collagen, type VI, alpha 1 | -1,74 |
| BE201395 | *zgc:103639* | MYLPF | fast skeletal myosin light chain 2 | -1,84 |
| BI865912 | *-* | S1PR1 | sphingosine-1-phosphate receptor 1 | -1,85 |
| BE693169 | *tnnt3b* | TNNT3 | troponin T type 3 (skeletal, fast) | -2,2 |
| BI840762 | *LOC794906* | GPM6A | glycoprotein M6A | -2,4 |
| AW058837 | *lrrn1* | LRRN1 | leucine rich repeat neuronal 1 | -2,46 |
