# Supplementary material for: Early Embryonic Gene Expression Profiling of Zebrafish Prion Protein (Prp2) Morphants
Source: PLoS One. 2010 Oct 22;5(10):e13573. doi: 10.1371/journal.pone.0013573 (PMC2962645; doi:10.1371/journal.pone.0013573)
Supplement: Table S3 — Fold change of differentially expressed genes which were not mapped by IPA in 24 hpf prp2-MO2 injected zebrafish embryos. These genes are annotated with zebrafish gene symbols obtained from the Genome Institute in Singapore. (0.15 MB DOC) [file pone.0013573.s006.doc]

Table S3: Fold change of differentially expressed genes which were not mapped by IPA in 24 hpf prp2-MO2 injected zebrafish embryos. These genes are annotated with zebrafish gene symbols obtained from the Genome Institute in Singapore.

| *GenBank* | *Zebrafish Gene Symbol* | *Unigene Descp* | *Fold change* |
| --- | --- | --- | --- |
| BE605628 | - |  | 5,77 |
| BI708483 | - | Transcribed locus, moderately similar to NP_035175.1 proliferating cell nuclear antigen [Mus musculus] | 4,99 |
| BM155067 | *zgc:136826* | Zgc:136826 | 4,1 |
| AW165150 | - |  | 2,87 |
| BI891065 | LOC559158 | Similar to LOC494737 protein | 2,58 |
| BI673285 | *zgc:73230* | Zgc:73230 | 2,25 |
| BI980224 | *wu:fa11b04* | Wu:fa11b04 | 2,1 |
| AA495040 | *wu:fa05f02* | Wu:fa05f02 | 1,92 |
| BG306206 | - | Transcribed locus | 1,87 |
| AI476962 | *wu:fb55h10* | Wu:fb55h10 | 1,8 |
| BI887495 | - | CDNA clone IMAGE:7000716, containing frame-shift errors | 1,79 |
| AI942866 | LOC100000419 | Hypothetical protein LOC100000419 | 1,77 |
| AW117056 | - | Transcribed locus | 1,76 |
| BI887138 | *wu:fc15e02* | Wu:fc15e02 | 1,69 |
| BM034693 | LOC566559 | Hypothetical LOC566559 | 1,68 |
| AI522349 | *wu:fb18g01* | Wu:fb18g01 | 1,68 |
| BM101524 | - | Transcribed locus | 1,66 |
| BI474299 | - | Transcribed locus | 1,65 |
| BQ285114 | *zgc:101724* | Zgc:101724 | 1,64 |
| BI325090 | *znfl1* | Zinc finger-like gene 1 | 1,63 |
| AI544649 | *wu:fb77b09* | Wu:fb77b09 | 1,62 |
| BE200552 | - | Transcribed locus | 1,58 |
| BI897147 | - |  | 1,58 |
| BI886699 | LOC100002850 | Similar to Secretory carrier membrane protein 2, like | 1,57 |
| BE017652 | LOC561592 | Hypothetical LOC561592 | 1,57 |
| BI842933 | MGC171577 | Hypothetical protein LOC100003142 | 1,57 |
| AI942952 | *wu:fc39c01* | Wu:fc39c01 | 1,53 |
| AW077940 | *wu:fj01d05* | Wu:fj01d05 | 1,53 |
| AW019428 | *zgc:64137* | Zgc:64137 | 1,51 |
| BI889280 | - | Transcribed locus | 1,5 |
| BM181246 | LOC568707 | Type IV antifreeze protein precursor | 1,49 |
| AI667325 | - | Transcribed locus, moderately similar to NP_060737.1 H2A histone family, member J isoform 1 [Homo sapiens] | 1,49 |
| AW281809 | - | Transcribed locus | 1,49 |
| BI882203 | - | Transcribed locus | 1,49 |
| BI892373 | - |  | 1,47 |
| BG306173 | *si:ch211-240l19.1* | Si:ch211-240l19.1 | 1,46 |
| AI641408 | *si:ch211-240l19.1* | Si:ch211-240l19.1 | 1,46 |
| BG307513 | *zgc:92153* | Zgc:92153 | 1,44 |
| BG305991 | - |  | 1,44 |
| AI330865 | - |  | 1,44 |
| BM071941 | *zgc:103624* | Zgc:103624 | 1,43 |
| AI641169 | *wu:fc20c08* | Wu:fc20c08 | 1,42 |
| BI879035 | *wu:fc47b11* | Wu:fc47b11 | 1,42 |
| BI980261 | *zgc:153795* | Zgc:153795 | 1,42 |
| AI721686 | *zgc:153970* | Zgc:153970 | 1,42 |
| AI545455 | - | Transcribed locus | 1,42 |
| AI943105 | *wu:fc47a08* | Wu:fc47a08 | 1,41 |
| AI384746 | - |  | 1,41 |
| BI896386 | *zgc:100829* | Zgc:100829 | 1,4 |
| AB055677 | - | D178 mRNA, 3'UTR, partial sequence | 1,39 |
| BI673276 | LOC567029 | Similar to ribosome binding protein 1 homolog 180kDa | 1,38 |
| BE017542 | - |  | 1,38 |
| BI879889 | - | Transcribed locus, strongly similar to NP_062686.1 ring-box 1 [Mus musculus] | 1,38 |
| AW077961 | *anxa1b* | Annexin A1b | 1,37 |
| AI943081 | *wu:fc84a08* | Wu:fc84a08 | 1,37 |
| AW420968 | - | Transcribed locus | 1,37 |
| AW466712 | - |  | 1,37 |
| AL720391 | LOC572235 | Hypothetical LOC572235 | 1,36 |
| AW128372 | *si:ch211-157p10.1* | Si:ch211-157p10.1 | 1,36 |
| AI883979 | *wu:fc68e03* | Wu:fc68e03 | 1,36 |
| BI890505 | - | Transcribed locus, strongly similar to XP_683970.2 PREDICTED: similar to LOC553461 protein [Danio rerio] | 1,36 |
| BI840692 | LOC100002247 | Hypothetical protein LOC100002247 | 1,35 |
| AI878441 | *wu:fc10a09* | Wu:fc10a09 | 1,35 |
| AI626449 | - |  | 1,35 |
| AI942960 | - |  | 1,35 |
| AI588147 | - |  | 1,35 |
| AI721935 | *zgc:77098* | Zgc:77098 | 1,34 |
| AI793723 | - | Transcribed locus | 1,34 |
| AL590150 | - |  | 1,34 |
| AI942585 | - | Transcribed locus | 1,34 |
| AI477020 | - | Transcribed locus | 1,34 |
| BI887770 | LOC559540 | Similar to conserved hypothetical protein | 1,33 |
| AW184205 | LOC565309 | Hypothetical LOC565309 | 1,33 |
| AW019729 | *wu:fd56d05* | Wu:fd56d05 | 1,33 |
| AI545423 | - |  | 1,33 |
| BM155225 | *zgc:92066* | Zgc:92066 | 1,32 |
| AI601390 | *wu:fc10f03* | Wu:fc10f03 | -1,32 |
| BG305515 | - | Transcribed locus | -1,32 |
| BI705759 | - |  | -1,32 |
| BM024150 | - | Transcribed locus | -1,32 |
| BM104048 | *phf23b* | PHD finger protein 23b | -1,33 |
| AI353083 | *hbae3* | Hemoglobin alpha embryonic-3 | -1,34 |
| BI845187 | *wu:fa12e08* | Wu:fa12e08 | -1,34 |
| AI522514 | *wu:fb20f08* | Wu:fb20f08 | -1,34 |
| BI325077 | *wu:fc45e06* | Wu:fc45e06 | -1,34 |
| BI563084 | *zgc:103663* | Zgc:103663 | -1,34 |
| AW116322 | *zgc:56036* | Zgc:56036 | -1,34 |
| AW175553 | - | Transcribed locus | -1,34 |
| AI558478 | *wu:fb79f11* | Wu:fb79f11 | -1,35 |
| BI880330 | - | Transcribed locus | -1,35 |
| AL591482 | - |  | -1,36 |
| U14590 | *ascl1b* | Achaete-scute complex-like 1b (Drosophila) | -1,37 |
| AF180891 | *mylz3* | Myosin, light polypeptide 3, skeletal muscle | -1,37 |
| AI626437 | *si:ch73-266o15.1* | Si:ch73-266o15.1 | -1,37 |
| BI846314 | *wu:fd23c12* | Wu:fd23c12 | -1,37 |
| BM095898 | *wu:fj35h06* | Wu:fj35h06 | -1,37 |
| AW076611 | - |  | -1,37 |
| BM102223 | - | Transcribed locus | -1,38 |
| AI588403 | *wu:fc37a05* | Wu:fc37a05 | -1,39 |
| BI865034 | *zgc:171560* | Zgc:171560 | -1,39 |
| AA495154 | *wu:fa04a07* | Wu:fa04a07 | -1,41 |
| AL718173 | *zgc:165530* | Zgc:165530 | -1,42 |
| BM026607 | - |  | -1,42 |
| BM183857 | LOC100007086 | Similar to aspartic acid-rich protein aspolin2-1 | -1,43 |
| AW343510 | - | Transcribed locus | -1,43 |
| AW420381 | LOC798299 | Hypothetical protein LOC798299 | -1,44 |
| AI964216 | *rps9* | Ribosomal protein S9 | -1,44 |
| BI880151 | *wu:fc51f04* | Wu:fc51f04 | -1,44 |
| AW154075 | *wu:fk35a11* | Wu:fk35a11 | -1,45 |
| AW117106 | *wu:fe26g04* | Wu:fe26g04 | -1,48 |
| BG883325 | LOC100001967 | Hypothetical protein LOC100001967 | -1,5 |
| AA495157 | *sb:cb319* | Sb:cb319 | -1,5 |
| AW203163 | *zgc:110586* | Zgc:110586 | -1,5 |
| AI478002 | - | Transcribed locus | -1,5 |
| AA605677 | *zgc:123210* | Zgc:123210 | -1,51 |
| AI522447 | - | Transcribed locus | -1,51 |
| BE200753 | - |  | -1,52 |
| BG985455 | *id:ibd1128* | Id:ibd1128 | -1,54 |
| BI891936 | *zgc:136942* | Zgc:136942 | -1,71 |
| BG728492 | - | Transcribed locus | -1,75 |
| BM155853 | *zgc:171791* | Zgc:171791 | -1,85 |
| BM154771 | *pvalb3* | Parvalbumin 3 | -1,87 |
| AF180888 | *pvalb2* | Parvalbumin 2 | -1,89 |
| CF569098 | *zgc:136942* | Zgc:136942 | -1,94 |
| BI533161 | *wu:fc46h12* | Wu:fc46h12 | -2 |
| AI722369 | - | Transcribed locus, weakly similar to XP_426745.1 PREDICTED: similar to hypothetical protein FLJ32214, partial [Gallus gallus] | -2,02 |
| AF116539 | *iclp2* | Invariant chain-like protein 2 | -2,06 |
| AI331606 | *wu:fa99d09* | Wu:fa99d09 | -2,22 |
| AI618133 | *pvalb1* | Parvalbumin 1 | -2,54 |
